# The role of BRCA1-IRIS in the development and progression of triple negative breast cancers in Egypt: possible link to disease early lesion

**DOI:** 10.1186/s12885-017-3283-8

**Published:** 2017-05-12

**Authors:** Danielle Bogan, Lucio Meile, Ahmed El Bastawisy, Hend F. Yousef, Abdel-Rahman N. Zekri, Abeer A. Bahnassy, Wael M. ElShamy

**Affiliations:** 1 Cancer Institute, University of Mississippi Medical Center, 2500 N. State Street, Jackson, MS 39216 USA; 2 Department of Genetics, Louisiana State University, Louisiana, USA; 3 Medical Oncology, National Cancer Institute, Cairo University, Cairo, Egypt; 4 Cytogenetics and Molecular Genetics, National Cancer Institute, Cairo University, Cairo, Egypt; 5 Virology and Immunology, National Cancer Institute, Cairo University, Cairo, Egypt; 6 Molecular Pathology, National Cancer Institute, Cairo University, Cairo, Egypt; 7 Present Address: San Diego Biomedical Research Institute, 10865 Road to Cure, Suite 100, San Diego, CA 92121 USA

**Keywords:** Breast cancer, Triple negative, BRCA1-IRIS, Metastasis, Egypt, Tumor-initiating cells, Breast cancer early lesion

## Abstract

**Background:**

Breast cancer is the most globally diagnosed female cancer, with the triple negative breast cancer (TNBC) being the most aggressive subtype of the disease. In this study we aimed at comparing the effect of BRCA1-IRIS overexpression on the clinico-pathological characteristics in breast cancer patients with TNBC or non-TNBC in the largest comprehensive cancer center in Egypt.

**Methods:**

To reach this goal, we conducted an observational study at the National Cancer Institute (NCI), Cairo University (Cairo, Egypt). The data on all diagnosed breast cancer patients, between 2009 and 2012, were reviewed. BRCA1-IRIS expression measured using real time RT/PCR in these patients’ tumor samples was correlated to tumor characteristics, such as to clinico-pathological features, therapeutic responses, and survival outcomes.

**Results:**

96 patients were enrolled and of these 45% were TNBC, and 55% were of other subtypes (hereafter, non-TNBC). All patients presented with invasive ductal carcinomas. No significant difference was observed for risk factors, such as age and menopausal status between the TNBC and the non-TNBC groups except after BRCA1-IRIS expression was factored in. The majority of the tumors in both groups were ≤5 cm at surgery (*p = 0.013*). However, in the TNBC group, ≤5 cm tumors were BRCA1-IRIS-overexpressing, whereas in the non-TNBC group they were BRCA1-IRIS-negative (*p = 0.00007*). Most of the TNBC patients diagnosed with grade 1 or 2 were BRCA1-IRIS-overexpressing, whereas non-TNBCs were IRIS-negative (*p = 0.00035*). No statistical significance was measured in patients diagnosed with grade 3 tumors. Statistically significant difference between TNBCs and non-TNBCs and tumor stage with regard to BRCA1-IRIS-overexpression was observed. Presence of axillary lymph node metastases was positively associated with BRCA1-IRIS overexpression in TNBC group, and with BRCA1-IRIS-negative status in the non-TNBC group (*p = 0.00009*). Relapse after chemotherapy (*p < 0.00001*), and local recurrence/distant metastasis after surgery (*p = 0.0028*) were more pronounced in TNBC patients with BRCA1-IRIS-overexpressing tumors compared to non-TNBC patients. Finally, decreased disease-free survival in TNBC/BRCA1-IRIS-overexpressing patients compared to TNBC/BRCA1-IRIS-negative patients, and decreased overall survival in TNBC as well as non-TNBC patients was driven by BRCA1-IRIS overexpression.

**Conclusions:**

TNBC/BRCA1-IRIS-overexpressing tumors are more aggressive than TNBC/BRCA1-IRIS-negative or non-TNBC/BRCA1-IRIS-overexpressing or both negative tumors. Further studies are warranted to define whether BRCA1-IRIS drives the early TNBC lesions growth and dissemination and whether it could be used as a diagnostic biomarker and/or therapeutic target for these lesions at an early stage setting.

## Background

Breast cancer is a globally common female malignancy accounting for 21% of all cancers [1]. Egypt is no exception with figures reaching 38% of all newly diagnosed cancer cases [2, 3]. Breast cancer is a heterogeneous disease composed of different molecular subtypes based on the gene expression profiling and the alterations exist in the genome [4, 5]. These subtypes have different clinico-pathological and molecular features that impact on the prognosis and treatment strategies [6]. “Triple negative” breast cancer (ERα-negative, PR-negative, HER2 not amplified) is itself a heterogeneous group of diseases [7]. Most of the work characterizing TNBC has focused on North American and European patients. We do not yet know to what extent the molecular features of TNBCs are conserved in different human populations. As a group, TNBC is characterized by aggressive clinical behavior, the younger age at diagnosis, early recurrence and with shorter disease-free survival [8]. In Egypt, the data regarding TNBC is sparse and inconclusive therefore, more studies describing the clinico-pathological features, prognostic biomarkers, and more importantly, therapeutic strategies are urgently needed [9, 10].

BRCA1-IRIS is a novel oncogene produced by an alternate usage of the well-known BRCA1 locus [11]. BRCA1-IRIS overexpression [12] drives expression of basal biomarkers, epithelial to mesenchymal transition (EMT)-inducers [13] and stemness-enforcers [14] in breast cancer cells. Since all are hallmarks of TNBCs, this led us to originally propose that BRCA1-IRIS overexpression drives the formation of TNBCs. In fact, BRCA1-IRIS overexpression correlates specifically with loss of BRCA1 expression in these tumors, another hallmark of TNBCs [12, 14]. BRCA1-IRIS overexpression also correlates with increased drug resistance in breast and ovarian cancer cells [15, 16]. BRCA1-IRIS inhibition using a novel inhibitory peptide sensitized triple negative breast cancer cells to paclitaxel treatment [13] and ovarian cancer cells to cisplatin treatment [17], in vitro and in vivo.

The prevailing view considers metastasis as the final step in cancer progression. Support of this view comes from clinical and experimental observations that show patients’ death from metastatic not primary disease, cure after an early surgery, accumulation of mutations during local progression [18], and repeated rounds of in vivo selection led to cell lines with increased metastasis formation [19–21]. Other clinical and experimental observations, however, support dissemination of metastatic precursors from early cancer lesions. For example; suppressing invasion using matrix-metalloproteinases inhibitors did not inhibit metastasis [22, 23], patients with poor prognosis can be identified by gene expression studies before manifestation of metastasis [24]. Although genetic predisposition seems to determine metastatic spread, knowing when exactly these metastatic precursors disseminate from primary tumors is critical for designing therapies that target them at this stage and prevent systemic cancer.

We previously addressed the issue of whether BRCA1-IRIS overexpression is indeed involved in early versus late metastatic spread by analyzing circulating tumor cells (CTCs) in peripheral blood and disseminated tumor cells (DTCs) in bone marrow of mice injected with dilution of BRCA1-IRIS overexpressing cells [14]. Injecting fewer rather than large number of such cells displayed increased capabilities to generate tumors, CTCs and DTCs, clearly support BRCA1-IRIS overexpressing TNBC cells early dissemination [14]. In the current study, we aimed at determining the prevalence of BRCA1-IRIS overexpression in a cohort of Egyptian patients with invasive breast cancers, defining the possible effect of BRCA1-IRIS overexpression on TNBCs clinical and biological behavior compared to the non-TNBCs, and whether its overexpression is associated with dissemination from early TNBC lesions.

## Methods

### Study cohort

Ninety-six breast cancer patients with primary invasive ductal carcinomas recently diagnosed and treated at the National Cancer Institute (NCI), Cairo University, (Cairo, Egypt) between September 2009 and October 2012 were included in the study [25, 26]. None of the patients showed metastasis at the time of initial diagnosis. Expression of ER, PR and HER-2/neu were assessed in all tumor samples. Based on this analysis 43 of the patients were negative for all three markers and thus considered TNBCs [mean age of 51.91 ± 12.34 SD, range: 30–78 years] and 53 showed expression of some/all of the markers and thus were considered non-TNBCs [mean age of 52.77 ± 12.13 SD, range: 27–81 years]. Twenty normal breast tissue samples obtained from reduction mammoplasty (mean age 35 ± 13.94 SD; range, 22–64 years) were included as controls in the study. WHO classification of breast tumors was used to grade the tumors and American Joint Committee on Cancer’s Staging Manual (7th edition) was used to stage the tumors [27, 28]. All participants signed written informed consent prior to enrollment in the study that was approved by the Institutional Review Board (IRB) of the NCI, Cairo, Egypt according to the 2011 Helsinki Declaration.

### Inclusion criteria

All study participants were 18 years or older. All patients presented with histologically-confirmed TNBC or non-TNBC breast cancer in accordance with the Eastern Cooperative Oncology Group (ECOG) Adequate performance: ≤2 [29]. All patients showed adequate hematological parameters including WBC count of ≥3.0 × 10^9^/l; ANC of ≥1.5 × 10^9^/l; platelet count of ≥100 × 10^9^/l; hemoglobin level of ≥9 g/l. Adequate liver function as shown by serum bilirubin of <1.5 × ULN; ALT and AST levels of <3 times normal values, and kidney function as shown by plasma creatinine level of <1.5 times normal value. Distant metastases were not observed in any of the patients at the time of diagnosis. Patients’ exclusion criteria were metastases within 1 month after surgery, pregnancy, breast-feeding, active second malignancy, or involvement in another clinical trial.

### Treatment and follow-up of patients

All patients received a follow-up that started immediately after surgery and lasted till death. Treatments: FEC100 as follows: 500 mg/m^2^ Cyclophosphamide (Baxter, Deerfield, IL, USA) intravenous infusion; 100 mg/m^2^ Epirubicin (Pfizer, New York, NY, USA) intravenous infusion 1; 500 mg/m^2^ Fluorouracil (Ebewe Pharm, Unterach, Austria) intravenous injection on day of adjuvant for three cycles, followed by 75 mg/m^2^ docetaxel (Sanofi-Aventis, Paris, France) for four cycles every 21 days with standard pre-medication (anti-emetics, anti-allergic medications and proton pump inhibitors). Radiotherapy when indicated (50Gy in 2Gy daily fractions) was followed by hormonal therapy also whenever indicated in ER and/or PR positive tumors. Response Evaluation Criteria in Solid Tumors (RECIST) was used to assess response to treatment. Complete response (CR) = patients who showed complete disappearance of disease confirmed at 4 weeks; partial response (PR) = patients who showed ≥30% reduction in tumor size at 4 weeks, stable disease (SD) = patients who showed neither CR nor PR at 4 weeks, and finally, progressive disease (PD) = patients who showed presence of metastasis and/or recurrence (observed as a 20% increase in tumor measurements or appearance of new lesions) at 4 weeks [30]. The median follow-up period was 33 months. Local recurrence and distant metastases were assessed; and disease-free survival (DFS) and overall survival (OS) were calculated.

### Pathology and immunohistochemistry

Immunohistochemistry for molecular markers done on tumor samples in the Department of Pathology, Cairo University, NCI. Nuclear expression of ER or PR proteins and membranous expression of the HER2 staining were detected according to protocols described earlier [31, 32]. In brief, deparaffinized, formalin-fixed tissues, were labeled with monoclonal mouse antibodies for ER and PR proteins (DAKO) [31], and the qualitative, FDA-approved clinical test “HerceptTest (DAKO) for Her2 [32], using automated immunostainer and following the manufacturer’s protocol [31]. Staining interpretation was according to ASCO/CAP guidelines. For ER/PR, ≤ 1% nuclear staining was considered negative score if normal adjacent mammary gland ductules were present in the section and served as an internal positive control. HER2 scoring was graded based on the degree and intensity of membrane labeling. A 0–3+ scale was adopted with 0 = no/faint/incomplete/barely detectable membrane labeling in <10% of tumor cells, 1^+^ = faint/incomplete/barely detectable membrane labeling in >10% of tumor cells, 2^+^ = incomplete/weak-moderate complete membrane labeling in >10% of tumor cells, or complete/intense membrane labeling in <10% of tumor cells, and 3^+^ = intense/complete membrane labeling in >10% of tumor cells. A score of 0 or 1+ was considered negative for HER2 expression, whereas a score of 2+ or 3+ was considered positive. Tumors negative for ER, PR and HER2/*neu* were classified as triple-negative breast cancer (TNBC).

### RNA extraction and quantitative real time PCR (qPCR)

RNA was extracted from normal or tumors samples using RNAeasy Mini Kit (Qiagen, Milan, Italy), according to the manufacturer’s instructions. RNA was first reverse-transcribed using iScriptTMcDNA Synthesis Kit (Bio-Rad, Milano, Italy), according to the manufacturer’s instructions. Amplification of BRCA1-IRIS mRNA in the samples was assessed in triplicates using the primers BRCA1-IRIS Forward: GTCTGAGTGACAAGGAATTGGTTT; and BRCA1-IRIS Reverse: TTAACTATACTTGGAAATTTGTAAAATGTG using the Syber Green technique according to manufacturer’s protocols (Applied Biosystems, Inc., Foster City, CA, USA). Expression in the samples was normalized against the expression of the house-keeping gene; β-actin using the Forward: ACAGAGCCTCGCCTTTGC; and Reverse: GCGGCGATATCATCATCC primers. The following was used to measure the relative level of BRCA1-IRIS mRNA in each sample. Mean Ct was calculated for each sample. ΔCt = Ct for BRCA1-IRIS - Ct for β-actin. The ΔΔCt = [(Ct for BRCA1-IRIS - Ct for β-actin) for sample A - (Ct for BRCA1-IRIS - Ct for β-actin for sample B)]. Statistical analysis used the ΔΔCt not the raw Ct data [33].

### Statistical analysis

Statistical analysis was performed using SPSS, version 20.0 (IBM SPSS, Armonk, NY, USA) and expressed as the mean rank or mean ± standard deviation for continuous variables. Chi Square (**χ**2) test was used to assess the association of TNBC or non-TNBC with other clinico-pathological variables. All *P*-values are two-tailed, where *P* < 0.05 was considered statistically significant. Kaplan-Meier analysis and curves was used for the associations with survival.

## Results

To explore BRCA1-IRIS role in TNBC development and progression in Egyptian breast cancer patients, BRCA1-IRIS expression was assessed in 96 samples obtained from patients’ who attended the clinics of the NCI, Cairo during the period from September 2009 and October 2012. Expression data were correlated to clinical, pathological, and survival data of these patients.

Based on hormone receptor status, 44.8% (43/96) of the patients were TNBCs and the remaining 55.2% (53/96) were allocated to other groups (i.e. ER^+^ and Her2-enriched, hereafter non-TNBCs), suggesting a higher prevalence of TNBCs among Egyptian patients compared to USA patients (~15%). Among the TNBC group, 65% (28/43) of the patients showed BRCA1-IRIS-overexpressing (with cutoff defined as expression ≥2fold compared to normal samples) tumors, while 35% (15/43) showed BRCA1-IRIS-negative (i.e. expressing levels similar to that observed in normal samples) tumors. By contrast, among the non-TNBCs patients, 28% (15/53) had BRCA1-IRIS-overexpressing tumors and 72% (38/53) had BRCA1-IRIS-negative tumors, suggesting that, similar to American patients, BRCA1-IRIS overexpression is more prevalent in TNBCs in an Egyptian population.

All patients mean age 52.38 ± 12.17 years (range: 27–81 years) did not differ significantly from TNBC patients mean age 51.91 ± 12.34 years, or non-TNBC patients mean age 52.77 ± 12.13 years (*p = 0.73*). However, although statistically insignificant, we observed that BRCA1-IRIS-overexpressing patients tended to be of younger age than BRCA1-IRIS-negative patients within the TNBC group (50.8 ± 13 vs. 54 ± 11.2 years), as well as the non-TNBC group (48 ± 9.5 vs. 55 ± 13 years). To accurately determine this notion a larger sample size is required.

Among the whole cohort, 46% (44/96) were premenopausal and 54% (52/96) were postmenopausal. In both the TNBC (24 vs. 19 patients) and the non-TNBC (28 vs. 25 patients) groups more postmenopausal than premenopausal patients was observed, although not statistically significant (Chi sq. 0.0851, *p = 0.77*, Table 1). In a univariate analysis comparing TNBCs and non-TNBCs for BRCA1-IRIS expression, there were statistically significant differences between BRCA1-IRIS-overexpressing and -negative tumors in the pre and postmenopausal patients. In the premenopausal group, more TNBC than non-TNBC patients presented with BRCA1-IRIS-overexpressing tumors, while more non-TNBC patients showed BRCA1-IRIS-negative tumors (Chi sq. 6.15, *p = 0.013*, Table 2). Similarly, in the postmenopausal group, more TNBC than non-TNBC patients showed BRCA1-IRIS-overexpressing tumors, while more non-TNBC patients showed BRCA1-IRIS-negative tumors (Chi sq. 7.44, *p = 0.006*, Table 2). Supporting the notion that BRCA1-IRIS overexpression is prevalent in pre- as well as post-menopausal Egyptian TNBC patients, suggesting that it could be an early event in the evolution of TNBCs.

**Table 1:** Comparison of tumor characteristics between TNBC and non-TNBC patients

	TNBC (*n* = 43) N (%)		Non-TNBC (*n* = 53) N (%)	χ2	*P*-value	Total
Menopausal status	Pre	19 (44%)	25 (47%)	0.0851	*p = 0.770*	44
	Post	24 (56%)	28 (53%)			52
Total		43	53			96
Tumor size	≤5 cm	23 (53%)	41(77%)	6.0869	*p = 0.013*	64
	>5 cm	20 (47%)	12 (23%)			32
Total		43	53			96
Grade	1 + 2	33 (77%)	47 (89%)	1.8674	*p = 0.171*	80
	3	8 (19%)	5 (9%)			13
Total		41	52			93
Stage	II	10 (23%)	29 (55%)	6.1913	*p = 0.012*	39
	III + IV	24 (56%)	22 (42%)			46
Total		34	51			85
Lymph-node	Present	33 (77%)	30 (57%)	4.2687	*p = 0.038*	63
	Absent	10 (23%)	23 (43%)			33
Total		43	53			96
Clinical response	CR	10 (23%)	41 (77%)	30.4317	*p < 0.00001*	51
	SD	2 (5%)	3 (7%)			5
	PD	31 (72%)	9 (17%)			40
Total		43	53			96
Relapse	Yes	27 (53%)	9 (23%)	25.8285	*p* < 0.000001	36
	No	10 (23%)	40%77%)			50
Total		37	49			86

*Abbreviations: TNBC* triple negative breast cancer, *CR* complete response, *SD* stable disease, *PD* progressive disease

**Table 2:** Comparison of clinico-pathological variables between TNBC and non-TNBC patients overexpressing or not BRCA1-IRIS (*aka* IRIS)

Menopausal	IRIS-positive		IRIS-negative		χ2	*P*-value	Total
Pre	TNBC	14 (74%)	TNBC	5 (26%)	6.15	*0.013*	19
	Non-TNBC	9 (36%)	Non-TNBC	16 (64%)			25
Post	TNBC	14 (58%)	TNBC	10 (42%)	7.44	*0.0064*	24
	Non-TNBC	6 (21%)	Non-TNBC	22 (79%)			28
Total		43		53			96
Tumor size	IRIS-positive		IRIS-negative		χ2	*P*-value	
≤5 cm	TNBC	21 (91%)	TNBC	2 (9%)	28.46	*<0.000001*	23
	Non-TNBC	9 (22%)	Non-TNBC	32 (78%)			41
>5 cm	TNBC	12 (60%)	TNBC	8 (40%)	0.31	*0.5809*	20
	Non-TNBC	6 (50%)	Non-TNBC	6 (50%)			12
Total		43		53			96
Grade	IRIS-positive		IRIS-negative		χ2	*P*-value	
1 + 2	TNBC	23 (70%)	TNBC	10 (30%)	13.84	*0.0002*	33
	Non-TNBC	13 (28%)	Non-TNBC	34 (72%)			47
3	TNBC	5 (62%)	TNBC	3 (38%)	0.63	*0.4285*	8
	Non-TNBC	2 (40%)	Non-TNBC	3 (60%)			5
Total		43		50			93
Stage	IRIS-positive		IRIS-negative		χ2	*P*-value	
II	TNBC	8 (80%)	TNBC	2 (20%)	7.25	*0.0071*	10
	Non-TNBC	9 (31%)	Non-TNBC	20 (69%)			29
III + IV	TNBC	16 (67%)	TNBC	8 (33%)	7.14	*0.0075*	24
	Non-TNBC	6 (27%)	Non-TNBC	16 (73%)			22
Total		39		46			85
Lymph-node	IRIS-positive		IRIS-negative		χ2	*P*-value	
Present	TNBC	25 (76%)	TNBC	8 (24%)	17.28	*<0.000001*	33
	Non-TNBC	7 (23%)	Non-TNBC	23 (77%)			30
Absent	TNBC	4 (40%)	TNBC	6 (60%)	0.29	*0.5922*	10
	Non-TNBC	7 (30%)	Non-TNBC	16 (70%)			23
Total		43		53			96

Significantly different mean tumor size at diagnosis was observed in the TNBC group (5.5 ± 4.1 cm) compared to the non-TNBCs group (3.8 ± 2.7 cm). However, in both TNBC and non-TNBC groups more patients had <5 cm than ≥5 cm in diameter tumors at diagnosis (Chi sq. 6.0869, *p = 0.013*, Table 1). Univariate analysis showed that there were statistically significant differences between BRCA1-IRIS-overexpressing and -negative tumors among the <5 cm and not ≥5 cm group. TNBC <5 cm tumors were more often BRCA1-IRIS-overexpressing, whereas non-TNBC <5 cm tumors were more often BRCA1-IRIS-negative (Chi sq. 15.67, *p = 0.00007*, Table 2). No statistically significant difference in BRCA-IRIS expression status in either group was observed (Chi sq. 0.41, *p = 0.52124*, Table 2). These data show that TNBC tumors in this cohort tend to be larger than non-TNBC tumors and BRCA1-IRIS is overexpressed more frequently (75%, Table 2) in TNBCs of smaller size. This is consistent with the hypothesis that BRCA1-IRIS overexpression might be involved in formation of TNBC early lesion.

Using Nottingham histological grading, it was observed that tumors in the TNBC and the non-TNBC groups were mostly grade 1 and 2 not grade 3 with no statistically significant difference detected (Chi sq. 1.8674, *p = 0.171*, Table 1). According to univariate analysis statistically significant differences between BRCA1-IRIS-overexpressing and -negative tumors among the grade 1 + 2 and not grade 3 tumors were observed. TNBC grade 1 + 2 tumors were more often BRCA1-IRIS-overexpressing, whereas non-TNBC grade 1 + 2 tumors were more often BRCA1-IRIS-negative (Chi sq. 12.77, *p = 0.00035*, Table 2). No statistically significant difference in grade 3 tumors and BRCA-IRIS expression status in either group (Chi sq. 0.42, *p = 0.42853*, Table 2). These data show that BRCA1-IRIS is overexpressed more frequently in low-grade TNBC (53%, Table 2). Again, reinforcing the hypothesis that BRCA1-IRIS overexpression might be involved in formation of TNBC early lesion.

In terms of AJCC tumor stage, the majority of tumors in the TNBC group were stage III + IV not II, whereas more non-TNBC tumors were stage II than III + IV (Chi sq. 6.1913, *p = 0.012*, Table 1). BRCA1-IRIS overexpressing stage II tumors were equally divided between TNBCs and non-TNBCs, whereas BRCA1-IRIS negative stage II tumors were often non-TNBC, which was statistically significant (Chi sq. 7.25, *p = 0.00709*, Table 2). Moreover, more stage III + IV TNBC tumors were BRCA1-IRIS overexpressing, whereas more stage III + IV non-TNBC tumors were BRCA1-IRIS-negative, which also was statistically significant (Chi sq. 6.50, *p = 0.01076*, Table 2). These data show that BRCA1-IRIS is overexpressed more frequently in higher stage TNBC tumors (39%, Table 2), suggesting that although low grade, BRCA1-IRIS overexpressing TNBC tumors are of higher stage implying increased aggressiveness in early BRCA1-IRIS overexpressing TNBC lesions.

Positive axillary lymph nodes (LN) metastasis was detected in 76.7% (33/43) of the TNBC patients compared to 56.6% (30/53) of the non-TNBC patients (Chi sq. 4.2687, *p = 0.038*, Table 1). Comparing the two groups for BRCA1-IRIS expression using univariate analysis showed that 73% (24/33) of node-positive TNBC tumors were BRCA1-IRIS-overexpressing, and 24% (8/33) were BRCA1-IRIS-negative, whereas only 27% (8/30) of the node-positive non-TNBC tumors were BRCA1-IRIS-overexpressing, and 77% (23/30) were BRCA1-IRIS-negative (Chi sq. 15.25, *p = 0.00009*, Table 2). Within the TNBC tumors, 40% (4/10) of node-negative cases were BRCA1-IRIS-overexpressing, while 60% (6/10) were BRCA1-IRIS-negative, whereas 30% (7/23) of node-negative non-TNBC were BRCA1-IRIS-overexpressing, and 70% (14/23) were BRCA1-IRIS-negative (Chi sq. 0.13, *p = 0.71686*, Table 2). These data show that lymph-node involvement is more prevalent in the TNBC than in the non-TNBC patients, and that BRCA1-IRIS overexpression is significantly more common in node-positive TNBC tumors group.

The majority of TNBC and the non-TNBC groups tumors were histologically invasive ductal carcinomas (IDC) regardless of BRCA1-IRIS status. This is the most common histological type in the US and Europe as well. The vast majority of the patients in both studied groups were treated with anthracycline-based chemotherapy for 3–7 months. More TNBCs patients showed progressive disease (PD) compared to complete response (CR) after therapy (31 vs. 10), whereas more non-TNBC patients showed CR compared to PD after therapy (41 vs. 9, Chi sq. 30.4317, *p* < 0.00001, Table 1). Among the 43 patients that had BRCA1-IRIS overexpressing tumors (Table 3), there was ﻿2 that showed ﻿stable disease (SD), both had non-TNBC tumors, there was 12 that showed CR, from those 33% (4/12) had TNBC tumors, and 67% (8/12) had non-TNBC tumors, and there was 29 that showed PD on therapy, from those 83% (24/29) had TNBC tumors, and only 17% (5/29) had non-TNBC tumors (Chi sq. 9.575, *p = 0.0020*, Table 3). In contrast, among the 53 patients that had BRCA1-IRIS negative tumors (Table 3), there was 5 patients that showed SD, from those 2 had TNBC tumors and 3 had non-TNBC tumors, there was 39 that showed CR, from those only 15% (6/39) had TNBC tumors, and 85% (33/39) had BRCA1-IRIS negative tumors, and there was 11 that showed PD on therapy, from those 64% (7/11) had TNBC tumors, while 36% (4/11) had non-TNBC tumors (Chi sq. 10.521, *p = 0.0052*, Table 3). These data suggest BRCA1-IRIS overexpression promotes chemotherapy resistance, especially in TNBC patients.

**Table 3:** Comparison of chemotherapy response in TNBC and non-TNBC patients overexpressing or not BRCA1-IRIS

Clinical response	CR	SD	PD	χ2	*p*-value	Total
IRIS^+^ TNBC Non-TNBC	12 4 8	2 0 2	29 24 5	9.575	*0.0020*	43
IRIS^−^ TNBC Non-TNBC	39 6 33	5 0 3	11 7 4	10.521	*0.0052*	53
Total	51	5	40			96

χ2 is Chi Square test, *Abbreviations:* *TNBC* triple negative breast cancer, *CR* complete response, *SD* stable disease, *PD* progressive disease

In this cohort, 44% (36/86, information about 10 patients was missing) of the patients relapsed and 58% (50/86) of the patients did not. Within the group that relapsed, 75% (27/36) had TNBC tumors, while 25% (9/36) had non-TNBC tumors. Within the group that did not relapse, 20% (10/50) had TNBC tumors, and 80% (40/50) had non-TNBC tumors (Chi sq. 25.8285, *p < 0.000001*, Table 1). We then compared these two groups for BRCA1-IRIS expression using univariate analysis. Among the BRCA1-IRIS-overexpresing patients (*n* = 38), 66% (25/38) relapsed and 34% (13/38) did not (Table 4). Among the 25 patients with BRCA1-IRIS-overexpressing tumors that relapsed, 80% (20/25) had TNBC tumors, and 20% (5/25) had non-TNBC tumors, while of the 13 patients with BRCA1-IRIS-overexpressing tumors that did not relapse, 36% (4/13) had TNBC tumors, and 64% (9/13) had non-TNBC tumors (Chi sq. 8.9, *p = 0.0028*, Table 4). Conversely, among patients with BRCA1-IRIS-negative tumors (*n* = 48), 23% (11/48) relapsed, and 77% (37/48) did not (Table 4). Seven out of the 11 (64%) BRCA1-IRIS-negative patients who relapsed had TNBC tumors, and only 4 (36%) had non-TNBC tumors, while among the 37 patients with BRCA1-IRIS-negative tumors that did not relapse, only 16% (6/37) had TNBC tumors, and 84% (31/37) had non-TNBC tumors (Chi sq. 9.66, *p = 0.0019*, Table 4). These data show increased relapse among TNBC patients when compared to non-TNBC patients, especially those overexpressing BRCA1-IRIS.

**Table 4:** Comparison of relapse in TNBC and non-TNBC patients overexpressing or not BRCA1-IRIS

Relapse	Yes	No	χ2	*p*-value	Total
IRIS^+^ TNBC Non-TNBC	25 20 5	13 4 9	8.90	*0.0028*	38
IRIS^−^ TNBC Non-TNBC	11 7 4	37 6 31	9.66	*0.0019*	48
Total	36	50			86

χ2 is Chi Square test

In the non-TNBC/BRCA1-IRIS-negative group, four patients showed distant metastasis to breast (*n* = 1), shoulder (*n* = 1), bone (*n* = 1) or lung (*n* = 1), and 5 patients of the non-TNBC/BRCA1-IRIS-overexpressing group showed metastasis to supraclavicular lymph nodes (*n* = 1), lung (*n* = 1), oral cavity (*n* = 1) and bone (*n* = 2). On the other hand, 7 patients in the TNBC/BRCA1-IRIS-negative group showed metastasis to bone (*n* = 1), brain (*n* = 1), bone + brain (*n* = 1), bone + breast (*n* = 2), and bone + liver (*n* = 2). Twenty patients in the TNBC/BRCA-1IRIS-overexpressing group showed metastasis to lung (*n* = 4), liver (*n* = 2), bone (*n* = 1), bone + liver (*n* = 4), bone + breast (*n* = 1), bone + lung (*n* = 1), bone + brain (*n* = 1), bone + liver + axilla (*n* = 6). These data show increased distant metastasis in TNBC compared to non-TNBC patients, particularly in cases with BRCA1-IRIS overexpression.

In non-TNBC (*n* = 53), the disease-free survival (DFS) was 29.13 ± 16.92 months compared to 18.67 ± 11.62 within the TNBC group (*n* = 43, *p = 0.001*, data not shown). DFS in non-TNBC patients with BRCA1-IRIS-negative tumors (*n* = 38) was 31.09 ± 16.98 months; while in the TNBC patients (*n* = 15) was 24.86 ± 10.93 months, which was not statistically significant (*p = 0.21*, not shown). However, DFS in patients with BRCA1-IRIS-overexpressing tumors in the non-TNBC group (*n* = 15) was 24.43 ± 16.36 months, while in the TNBC group (*n* = 28) DFS was 15.57 ± 10.85 months, which was statistically significant (*p = 0.039*, not shown).

Similarly, the overall survival (OS) within the non-TNBC group (*n* = 53) was 33.34 ± 15.40 months compared to 22.83 ± 12.24 within the TNBC group (*n* = 43, *p = 0.0005*, not shown). Among patients with BRCA1-IRIS-negative tumors in the non-TNBC group (*n* = 38), OS was 34.04 ± 16.37 months, while in the TNBC group (*n* = 15) OS was 28.10 ± 11.20 months, which was not statistically significant (*p = 0.21*, not shown). On the other hand, in patients with BRCA1-IRIS-overexpressing tumors, OS in the non-TNBC group (*n* = 15) was 31.64 ± 13.17 months, while in the TNBC group (*n* = 28) it was 20.20 ± 12.07 months, which was statistically significant (*p = 0.006*, not shown).

**Fig. 1:**
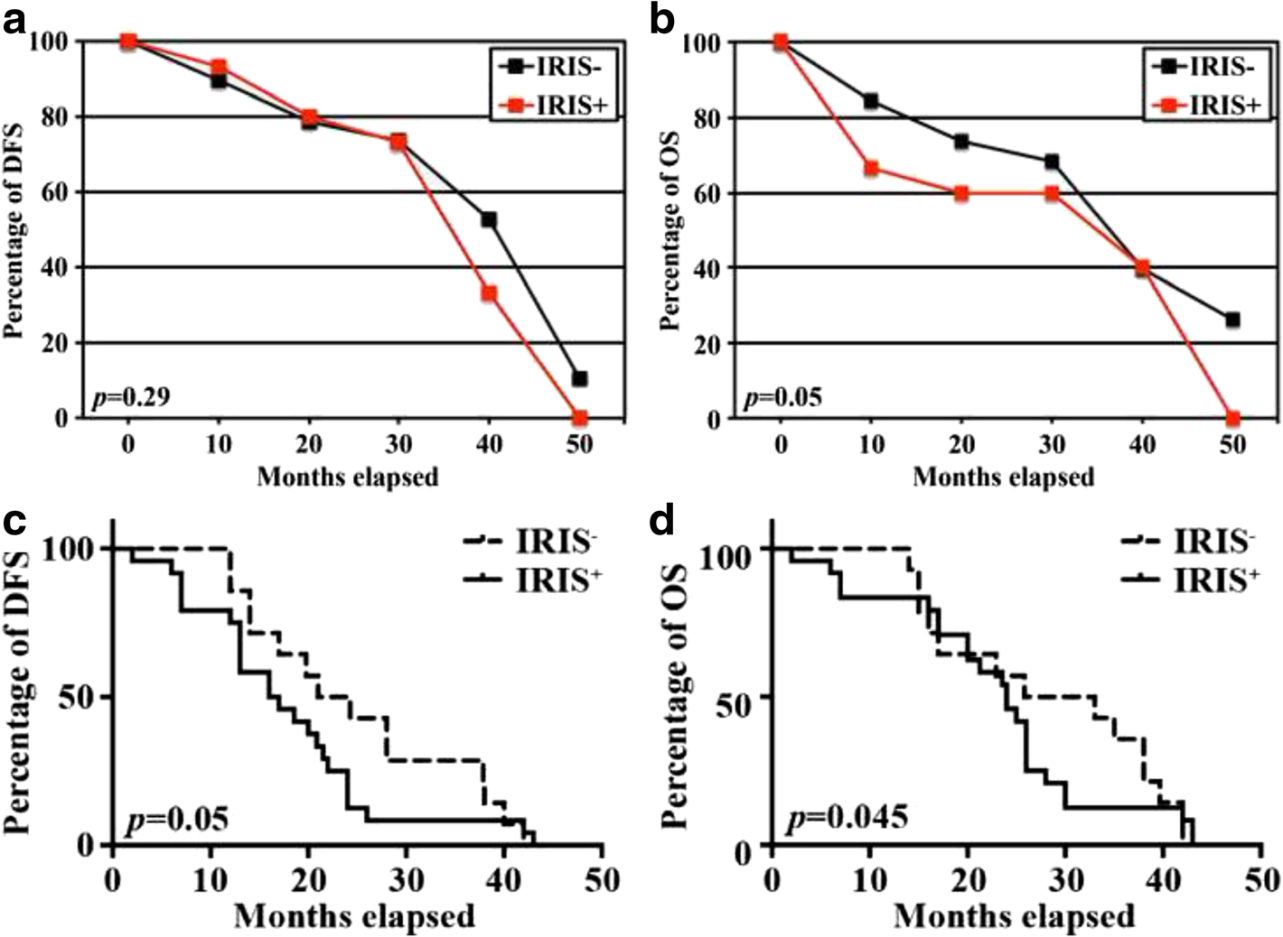
The effect of BRCA1-IRIS overexpression on DFS and OS in non-TNBC and TNBC patients. (**a**) No significant differences in DFS between BRCA1-IRIS-overexpressing and -negative tumors in the non-TNBC group (*p = 0.29*). (**b**) Statistically significant difference in OS between BRCA1-IRIS-overexpressing and -negative tumors in the non-TNBC group (*p = 0.05*, **b**). Significantly lower DFS (*p = 0.05*, **c**) and OS (*p = 0.045*, **d**) in BRCA1-IRIS-overexpressing tumors compared to BRCA1-IRIS-negative tumors in the TNBC group

As mentioned above, in the non-TNBC group DFS was not significantly different between BRCA1-IRIS-overexpressing or negative tumors (*p = 0.29*, Fig. 1a), whereas in the same group, OS was statistically different between BRCA1-IRIS-overexpressing and negative tumors (*p = 0.05*, Fig. 1b). On the other hand, in the TNBC group BRCA1-IRIS-overexpressing tumors had significantly lower DFS (*p = 0.05*, Fig. 1c) and OS (*p = 0.045*, Fig. 1d) than BRCA1-IRIS-negative tumors.

All patients were followed up for 50 months after diagnosis. Data were available for all except 2 non-TNBC/BRCA1-IRIS-negative patients (i.e. *n* = 36). In this group, 72% (26/36) of patients were alive at 50 months, and 28% (10/36) had died. Within the non-TNBC/BRCA1-IRIS-overexpressing group (*n* = 15), 67% (10/15) were alive at 50 months, and 33% (5/15) died. By contrast, among TNBC patients with BRCA1-IRIS-negative tumors (*n* = 15), 53% (8/15) were alive, and 47% (7/15) died by 50 months, whereas among TNBC patients with BRCA1-IRIS-overexpressing tumors (*n* = 28), 29% (8/28) were alive, while 71% (20/28) died by 50 months. These data show reduced DFS and OS among TNBC patients compared to non-TNBC patients that strongly correlate to BRCA1-IRIS overexpression. Thus BRCA1-IRIS drives poor survival outcomes in TNBC patients.

## Discussion

Like the rest of the world, Egypt suffers from an increased breast cancer burden. TNBC is an aggressive subgroup for which targeted therapies are lacking. Therefore, an urgent need exists for new effective therapeutic strategies with reduced toxicity. In the USA, TNBC accounts for ~15% of all breast cancer cases, with increased frequencies and with worst prognoses in young African American women [34–36]. In the present study, although the Egyptian cohort tested was small, the prevalence of TNBC was higher than the American at ~45%, but consistent with two previously published studies from Egypt [37, 38]. Furthermore, in the current study we showed that similar to USA population, BRCA1-IRIS is commonly overexpressed in TNBC compared to non-TNBC tumors in Egypt as well. However, compared to our previous study conducted in an American cohort [12], the overall percentage of BRCA1-IRIS overexpression in the TNBC cohort from Egypt (65%) was lower than that reported in the American cohort (88%) [12]. Similar studies are required to assess the frequency more accurately.

The current observational study was conducted to define the biological and pathological characteristics of TNBC tumors with BRCA1-IRIS overexpression [11]. The data show BRCA1-IRIS overexpression associates with lymph node and distant metastases, as well as poor clinical outcomes in TNBC patients among Egyptian patients. The long-term aim of the present study is to explore the use of BRCA1-IRIS overexpression as a predictive biomarker for TNBCs in Egyptian breast cancer patients, and to determine its potential usefulness as a therapeutic target [11, 12]. Furthermore, in our study, we found that 77% of the TNBC patients vs. 57% of non-TNBC patients showed involvement of the axillary lymph nodes (Table 1), significantly higher percentage of the TNBC group had BRCA1-IRIS-overexpressing tumors (Chi sq. 15.25, *p = 0.00009*, Table 2). Additionally, relapse was far more common in TNBC than non-TNBC cases. Indeed, among non-TNBC patients 16.9% (9/53) relapsed, versus 62.8% (27/43) in the TNBC group. A majority of relapsed patients in the TNBC group (20/27; 74%) were BRCA1-IRIS-overexpressing. Moreover, higher BRCA1-IRIS expression was associated with worse prognosis, and poorer outcomes after standard chemotherapy in Egyptian patients with TNBC, and lymph node and distant metastasis, and therefore higher AJCC stage. This is likely to explain the association of BRCA1-IRIS with poorer DFS and OS. Based on the current study and our previous study [14], we propose that BRCA1-IRIS overexpression contributes to the pathogenesis of TNBC and promotes its metastatic potential.

In this study, we showed that TNBC is a highly prevalent tumor type in Egyptian breast cancer patients, accounting for ~45% of all breast cancers in our study. Consistent with TNBC presentations in other populations, these tumors tended to have higher stage, larger size, earlier local and distant recurrences, and poorer disease outcome. The prevalence of BRCA1-IRIS overexpression amongst the Egyptian TNBC patients compared to the non-TNBC patients (65% vs. 28%) strongly suggests a tumor-promoting role, while its association with node and distant metastasis suggests metastatic driver role as well in TNBC patients.

Metastatic cancer remains the lethal clinical challenge in breast cancer. At diagnosis, prognostic factors are usually used to assess whether primary tumors have already disseminated or not. The prevailing model suggests that metastatic capacity is a late acquired event in tumorigenesis [39]. A new view, however, challenges this perception and proposes that breast cancer is intrinsically systemic disease that could disseminate at early stage while primary tumor is forming. These metastasis precursors are proposed to be a small sub-population of the tumor that show the most aggressive traits. For example, they are tumor-initiating cells (TICs) that underwent epithelial to mesenchymal transition (EMT). Identifying such disseminating-capable TICs and therapeutically targeting them most likely will prevent cancer progression. In the current study and others [14], several lines of evidence support BRCA1-IRIS overexpression as driver for the generation of such dissemination-capable TNBC cell. First, BRCA1-IRIS overexpression was associated with smaller tumor size. Second, although these tumors were histological low grade, they were of an advance stage. Third, BRCA1-IRIS-overexpressing/TNBC tumors showed prevalence to lymph-node and distant metastasis, low DFS and OS as well as an inherent chemotherapy-resistance. Forth, we recently showed that BRCA1-IRIS overexpression in fact initiates and maintains the tumor initiating phenotype in breast cancer cells [14]. If true, inhibiting BRCA1-IRIS-activity most likely could prevent metastatic precursors dissemination from early TNBC lesions and killing the patients.

## Conclusions

TNBC/BRCA1-IRIS-overexpressing tumors are more aggressive than TNBC/BRCA1-IRIS-negative or non-TNBC/BRCA1-IRIS-overexpressing or both negative tumors. Further studies are warranted to define whether BRCA1-IRIS drives the early TNBC lesions growth and dissemination and whether it could be used as a diagnostic biomarker and/or therapeutic target for these lesions at an early stage setting.
